# Comparison of the effectiveness, safety, and costs of anti‐Parkinson drugs: A multiple‐center retrospective study

**DOI:** 10.1111/cns.14531

**Published:** 2023-11-20

**Authors:** Wenting Li, Hua Zhang, Yuan Zhang, Ke Wang, Jiaojiao Hui, Zhanmiao Yi

**Affiliations:** ^1^ Department of Pharmacy Peking University Third Hospital Beijing China; ^2^ Department of Pharmacy, The Eighth Affiliated Hospital Sun Yat‐Sen University Shenzhen China; ^3^ Research Center of Clinical Epidemiology Peking University Third Hospital Beijing China; ^4^ Department of Health Research Methods, Evidence, and Impact McMaster University Hamilton Ontario Canada

**Keywords:** anti‐Parkinson drugs, economics, efficacy, levodopa equivalent dose, safety

## Abstract

**Aims:**

This study aimed to systematically compare the effectiveness, safety, and costs of different anti‐Parkinson drugs (APDs).

**Methods:**

This is a multi‐center study that retrospectively analyzed the data of 8420 outpatients with PD from 2014 to 2019 across 30 tertiary hospitals in China. The effectiveness was evaluated by changes in total dosages of APDs, normalized by levodopa equivalent dose (LED) and presented as ΔLEDs; levodopa equivalent dose cost (LEDc) represented the daily cost of APDs; and newly added diagnostics were represented as APDs‐related adverse events.

**Results:**

A total of 384 patients with eligible medical records for three consecutive years were enrolled. Patients treated with carbidopa/levodopa or levodopa/benserazide had significantly lower mean ΔLEDs than other groups (*p* < 0.01), followed by pramipexole and selegiline. The piribedil group had the highest ΔLEDs, with mean differences of 112.56–355.04 mg compared to other groups (*p* < 0.01). Meanwhile, LEDc in the levodopa/benserazide, carbidopa/levodopa, and piribedil groups were significantly lower than those in pramipexole or selegiline groups ($0.088–0.135/day for levodopa/benserazide; $0.070–0.126/day for carbidopa/levodopa; $0.112–0.138/day for piribedil; $0.290–0.332/day for pramipexole; $0.229–0.544/day for selegiline; *p* < 0.01). Patients with piribedil had more adverse events, with an incidence rate of 35.7%, followed by levodopa/benserazide (25.6%), selegiline (23.5%), carbidopa/levodopa (23.3%), and pramipexole (16.4%). Pramipexole showed a lower incidence rate of adverse events than piribedil, including neuropsychiatric symptoms (*p* = 0.006), headache/dizziness (*p* = 0.016), and gastrointestinal symptoms (*p* = 0.031).

**Conclusions:**

Carbidopa/levodopa or levodopa/benserazide might exhibit better clinical improvement with less medical cost, while piribedil presented less clinical improvement but a higher risk of headache/dizziness, gastrointestinal, and neuropsychiatric symptoms.

## Graphical abstract

With a lack of comprehensive comparisons among anti‐Parkinson drugs (APDs), this multi‐center retrospective study systematically compared the effectiveness, safety, and costs of different APDs. Carbidopa/levodopa or levodopa/benserazide might exhibit better clinical improvement with less medical cost, while piribedil presented less clinical improvement but a higher risk in headache/dizziness, gastrointestinal, and neuropsychiatric symptoms.

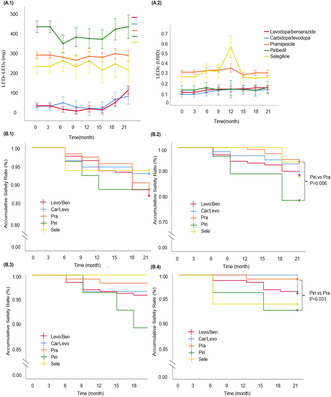

## 1 INTRODUCTION

Parkinson's disease (PD) is one of the most common neurodegenerative diseases and is clinically defined by symptoms including bradykinesia, tremor, rigidity, and postural instability.^1^ Globally, it was estimated that approximate 6.1 million people suffered from PD in 2016, and that number would increase to 8.7–9.3 million by 2030.^2^, ^3^, ^4^ In China, about 1.7% of people above 65 years old have PD.^5^ Additionally, PD is also characterized by its high mortality, and the mean survival time mostly ranges from 6.9 to 14.3 years.^6^, ^7^, ^8^ The PD disease course varies considerably, with those diagnosed early living longer than those diagnosed later in life.^9^ Thus, early diagnosis and intervention are critical for the PD treatment strategy before irreversible neuronal damage occurs.

Currently, the major pharmacotherapies for PD are dopamine precursors, dopamine agonists (DAs), monoamine oxidase‐B inhibitors (MAOBIs), catechol‐o‐methyl‐transferase inhibitors (COMTIs), anticholinergics, and antiglutamatergics. The different mechanisms of anti‐Parkinson drugs (APDs) might result in different effects and clinical applications. Levodopa‐based preparations, as a complement of dopamine, exhibited more than 50% improvement in symptomatic control lasting for 2–3 years.^10^, ^11^ However, long‐term levodopa‐based treatment might lead to motor complications, such as motor fluctuations and dyskinesia.^12^ Meanwhile, there is a considerable subgroup of nontypical PD patients who become progressively resistant to levodopa over time.^13^ DAs and MAOBIs are commonly used as adjuvant therapy to levodopa for alleviating motor complications, and also work well as monotherapy in controlling motor fluctuations for PD in the early stages.^14^, ^15^ Those therapies reduced off‐time and levodopa dose, and improved UPDRS scores in PD patients with levodopa‐induced motor complications, whereas they increased dyskinesia and numerous other adverse events.^14^ Moreover, the difficult distinction between motor complications caused by “overdose” or “sub‐dosage” makes it harder to choose the suitable medication. Due to the lack of direct head‐to‐head randomized controlled trials comparing the impacts of different APDs on the life quality of PD patients, understanding the effectiveness of different APDs is vital to optimize clinical decisions for PD treatment.

Potential risks or side effects should also be taken into account when selecting the optimal PD treatment strategy. For example, dopaminergic medication may increase motor complication risks and worsen some non‐motor manifestations (such as orthostatic hypotension and psychosis), which would limit its clinical application.^16^ Indirect comparison‐based researches suggested that DAs might be more effective than COMTIs and MAOBIs therapy,^14^ whereas DAs were associated with a higher overall incidence of adverse events than MAOBIs.^15^ More than 40% of individuals treated with DAs suffer from impulse control disorders (such as gambling and compulsive spending).^17^ Ultimately, most patients use multiple medications to attain complementary benefits and reduce dose‐related adverse events.^18^ Nonetheless, because current studies lack a safety comparison of existing APDs, uncertainty remains regarding the balance between the risks and benefits of these different APDs. Exploring the safety of all classes of APDs might provide critical information for selecting the safer therapy.

As a major contributor to the socioeconomic burden of PD, the costs of APDs might influence clinical decision‐making. In the US, the total direct and indirect costs for PD added up to $51.9 billion in 2017, of which medication was a primary source of direct costs.^19^ In Australia, medication expenses contributed approximately 35% of total health system costs related to PD, followed by general practitioner or specialist medical expenses at about 28%.^20^ In China, as the most costly component, medication costs accounted for 63% of the total direct medical cost to patients with PD in 2014, varying from 66% to 76% at different disease phases.^21^, ^22^ The cost of drug therapy might depend upon motor symptom severity, motor fluctuations, and PD subtype.^23^ Given the substantial expenditures, the cost‐effectiveness of APDs is of great importance to clinical decision‐making. However, current pharmacoeconomic studies on APDs mainly focus on the total drug cost without presenting medication utilization or comparing the different medications for PD cost. To solve these, we have first defined a new evaluation tool of levodopa equivalent dose cost (LEDc) and applied it to single‐center research,^24^ which could facilitate revealing the medication utilization and costs among different APDs.

Thus, a multi‐center study was conducted on outpatients from three perspectives: (1) to assess the effectiveness of different APDs through examining the changes in levodopa equivalent dose (LED) cumulative values; (2) to determine the safety via comparing the additions of other complications or diagnoses and other non‐anti‐PD drugs; (3) and to evaluate the cost‐effectiveness by calculating LEDc.

## 2 METHODS

### 2.1 Study centers and patients

Data of outpatient prescriptions were collected and analyzed from electronic medical records across 30 tertiary hospitals in China from 2014 to 2019. This study was part of a program approved by the Institutional Review Board of Beijing Tiantan Hospital, Capital Medical University (No. KY 2020‐077‐01).

Patients who met the following inclusion criteria were eligible for this study: (1) clearly diagnosed as PD; (2) age ≥18 years old; (3) patients with medical records for three consecutive years; (4) and patients with at least two clinic visits per year. We excluded patients who only took entacapone as the initial PD treatment.

### 2.2 Data collection and management

Data were gathered from the prescriptions of each outpatient visit, including administrative information (such as the date, specialty, and type of health insurance), demographics (such as age and gender), disease diagnoses, and clinical characteristics (such as each drug's brand or generic name, dosage, and unit cost). There was no missing data for the above items.

The consecutive 3‐year research period of each patient was divided into 12 intervals, each of which lasted for 3 months as a time node. The data of prescriptions within each time note were collocated, supposed to yield a total of 12 datasets for each outpatient. For those vacant time nodes without medical records, the data were imputed with the closest last available data, assuming that patients maintained the original therapy until they returned to visit. The initial medical data of each outpatient was obtained at the starting time point, which was the first visit within three consecutive years.

### 2.3 Outcome measures

#### 2.3.1 Effectiveness

The primary outcome was the long‐term effectiveness of the levodopa‐based preparations (levodopa/benserazide and carbidopa/levodopa), DAs (pramipexole and piribedil), and MAOBIs (selegiline). To facilitate the comparisons of different APDs, the total amounts of prescribed APDs were quantified and normalized by LED with corresponding conversion factors.^25^ Considering the failure of amantadine for conversion into LED or LEDc and the limited sample size, we excluded amantadine in this study. Effectiveness was defined as the scale and speed of LED cumulative dosage changes by calculating the gaps in LED cumulative values between subsequent time nodes (LED_n_) and the starting time node (LED_0_), using the following formula:

$$\text{ΔLED}={\mathrm{LED}}_{n}-{\mathrm{LED}}_{0}\;\left(n:\text{the number of time nodes},\text{from}\;0\;\text{to}\;7\right)$$

As the sample size decreased dramatically from the 24‐month period due to the loss of follow‐up, the follow‐up time for data analysis was considered ranging to 21 months in this study, namely at nodes 0–7.

#### 2.3.2 Safety

The second outcome was the long‐term safety of different APDs. The safety was deduced from the newly emerging complications and concomitant medications, which could represent the potential adverse events of APDs, mainly including gastrointestinal symptoms, neuropsychiatric symptoms, headache/dizziness, and abnormal blood pressure. Referring to the first visit within three consecutive years as the starting point, additional diagnosis, non‐anti‐PD drugs, and relative emerging timepoints for each outpatient were documented in detail to evaluate and compare the long‐term safety of different APDs.

#### 2.3.3 Costs

The third outcome was the drug costs of different APDs. Corresponding to the LED, LEDc was also applied to calculate and represent the daily cost of APDs transferring to levodopa equivalent dose, which was established and well‐validated in our previous article, the formulae as below.^24^ APD costs were defined as the values of LEDc for each outpatient within the corresponding time nodes.

$$\text{LEDc}\left(\mathrm{USD}/\mathrm{day}\right)=\frac{\text{Total costs of APDs}\left(\mathrm{USD}\right)}{\text{LEDs}\left(\mathrm{day}\right)}$$

### 2.4 Statistical analysis

The statistical analyses were performed with SPSS software version 27.0 (IBM Corp., Armonk, NY, United States). Baseline characteristics in each group were compared. Kolmogorov–Smirnov tests were performed to assess the distribution of continuous data. For quantitative data following a normal distribution, the mean with standard deviations (mean ± SD) or with standard error of the mean (mean ± SEM) was calculated and tested using the one‐way analysis of variance (ANOVA); for non‐normally distributed data, we calculated the median with an interquartile range [median (IQR)] and tested using the Kruskal–Wallis test; categorical data were presented as numbers (percentages) and tested using the Chi‐squared test. Repeated measures analysis of variance was used to investigate treatment differences and identify intergroup differences for effectiveness and cost outcomes. Meanwhile, the safety of different APDs was evaluated by survival analysis. The Kaplan–Meier method was used to calculate adverse reaction‐free events and overall incidences of adverse events, and the log‐rank (Mantel–Cox) test was used to compare different APD treatment groups.

## 3 RESULTS

### 3.1 Patient characteristics and anti‐Parkinson therapy profile

**FIGURE 1:**
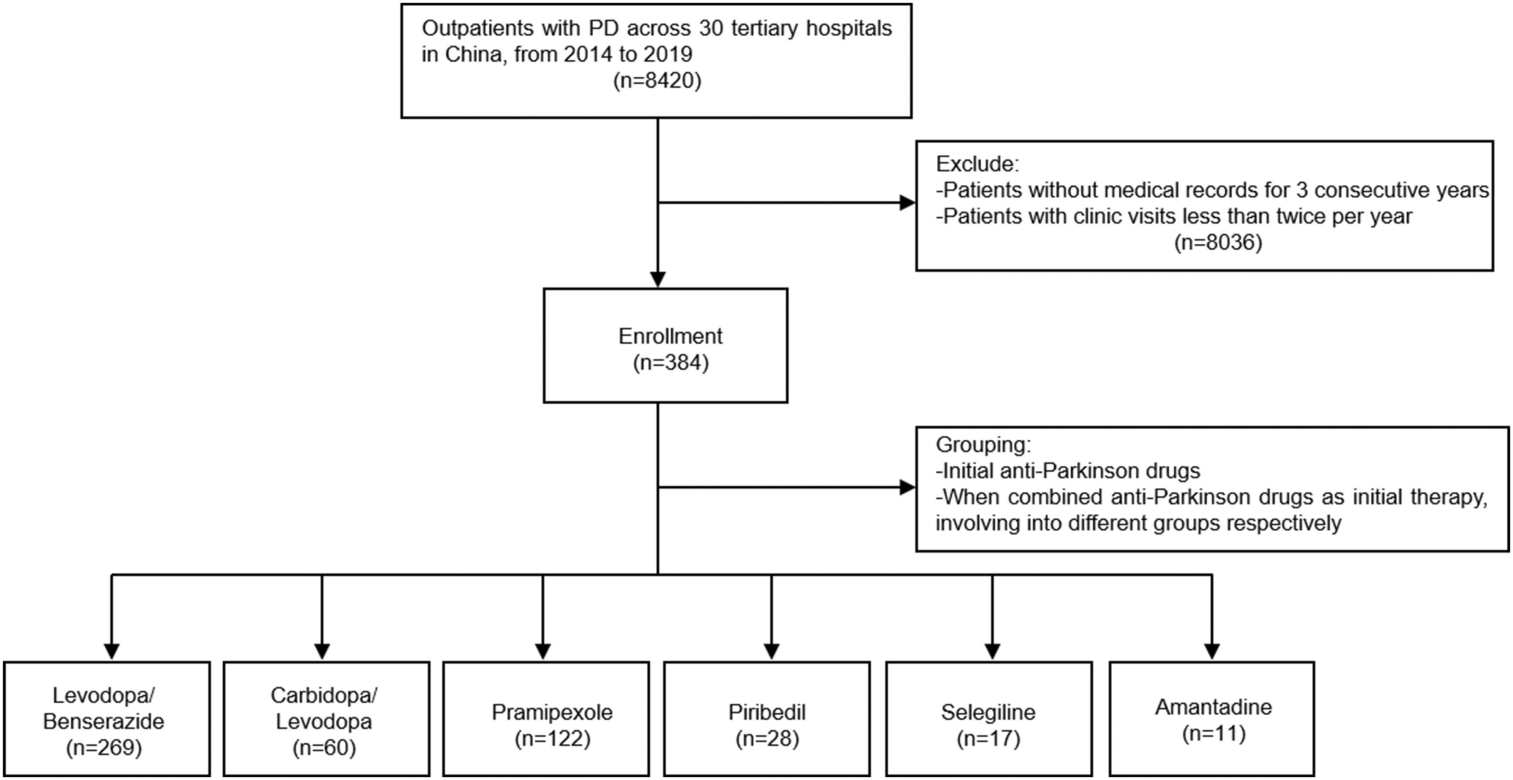
Flow chart of Parkinson's disease outpatient inclusion and grouping in this study.

A total of 384 patients with PD were screened from 8420 enrolled outpatients. Participants were divided into six groups depending on the initial APDs, including levodopa/benserazide (*n* = 269), carbidopa/levodopa (*n* = 60), pramipexole (*n* = 122), piribedil (*n* = 28), selegiline (*n* = 17), and amantadine (*n* = 11), among which single and combined APDs regimens were both included (Figure 1). For an overall baseline comparison, there was no statistically significant difference in sex and age among six groups (*p* > 0.05), while significant differences were found in initial LEDs and LEDc, which were corrected by calculating the gap values and comparing the change trends among intergroups in the following data analysis.

Included patients aged 72.11 ± 10.39 (mean ± SD) years old, and males accounted for 52.6% (Table 1). The PD patients largely consisted of seniors (>60 years), and the proportion was significantly higher than those below 60 years (89.3% vs. 10.8%, *p* < 0.001). For the anti‐Parkinson therapy, the average initial LEDs were 433.70 mg, with a high proportion (58.8%) of patients below 500 mg, indicating the relatively early clinical stage of PD for most recruited patients. Correspondingly, most patients have lower initial costs of APDs, with a mean initial LEDc of $0.141 per day, and over 95% of patients have an initial LEDc less than $0.435 per day.

**TABLE 1:** Baseline demographic and clinical characteristics of the included patients.

Characteristics	Levodopa/benserazide	Carbidopa/levodopa	Pramipexole	Piribedil	Selegiline	Amantadine	Overall
	Number (percentage, %)						
Gender							
Men	150 (55.8%)	25 (41.7%)	70 (57.4%)	13 (46.4%)	7 (41.2%)	5 (45.5%)	202 (52.6%)
Women	119 (44.2%)	35 (58.3%)	52 (42.6%)	15 (53.6%)	10 (58.8%)	6 (54.5%)	182 (47.4%)
Age (mean ± SD)	72.81 ± 10.44	73.02 ± 9.85	70.01 ± 9.99	74.18 ± 11.32	71.35 ± 10	69 ± 10.35	72.11 ± 10.39
<50	8 (3.0%)	2 (3.3%)	6 (4.9%)	2 (7.1%)	0 (0.0%)	1 (9.1%)	13 (3.4%)
50–59	21 (7.8%)	2 (3.3%)	10 (8.2%)	1 (3.6%)	2 (11.8%)	1 (9.1%)	28 (7.4%)
60–69	71 (26.4%)	17 (28.3%)	40 (32.8%)	5 (17.9%)	6 (35.3%)	2 (18.2%)	105 (27.3%)
70–79	89 (33.1%)	23 (38.3%)	48 (39.3%)	10 (35.7%)	4 (23.5%)	6 (54.5%)	131 (34.1%)
≥80	80 (29.7%)	16 (26.7%)	18 (14.8%)	10 (35.7%)	5 (29.4%)	1 (9.1%)	107 (27.9%)
Initial LEDs (mean ± SD, mg)	498.59 ± 254.04	420.94 ± 144.36	361.33 ± 241.94	562.89 ± 277.5	326.91 ± 234.56	605.68 ± 332.23	433.70 ± 256.56
<500 mg	132 (49.1%)	40 (66.7%)	84 (68.9%)	10 (35.7%)	12 (70.6%)	5 (45.5%)	226 (58.8%)
500–1000 mg	130 (48.3%)	20 (33.3%)	38 (31.1%)	18 (64.3%)	5 (29.4%)	4 (36.4%)	151 (39.3%)
>1000 mg	7 (2.6%)	0 (0.0%)	0 (0.0%)	0 (0.0%)	0 (0.0%)	2 (18.2%)	7 (1.8%)
Initial LEDc (mean ± SD, USD)	0.088 ± 0.087	0.070 ± 0.046	0.290 ± 0.420	0.112 ± 0.144	0.245 ± 0.230	0.042 ± 0.042	0.141 ± 0.268
<$0.435/day	266 (98.9%)	60 (100.0%)	110 (90.2%)	27 (96.4%)	13 (76.5%)	11 (100.0%)	368 (95.8%)
$0.435–0.725/day	2 (0.7%)	0 (0.0%)	5 (4.1%)	0 (0.0%)	3 (17.6%)	0 (0.0%)	8 (2.1%)
>$0.725/day	1 (0.4%)	0 (0.0%)	7 (5.7%)	1 (3.6%)	1 (5.9%)	0 (0.0%)	8 (2.1%)

*Note*: In total, 384 patients with Parkinson's disease were included and separated into six groups, with single and combined anti‐Parkinson drugs regimens.

### 3.2 Effectiveness outcomes

**FIGURE 2:**
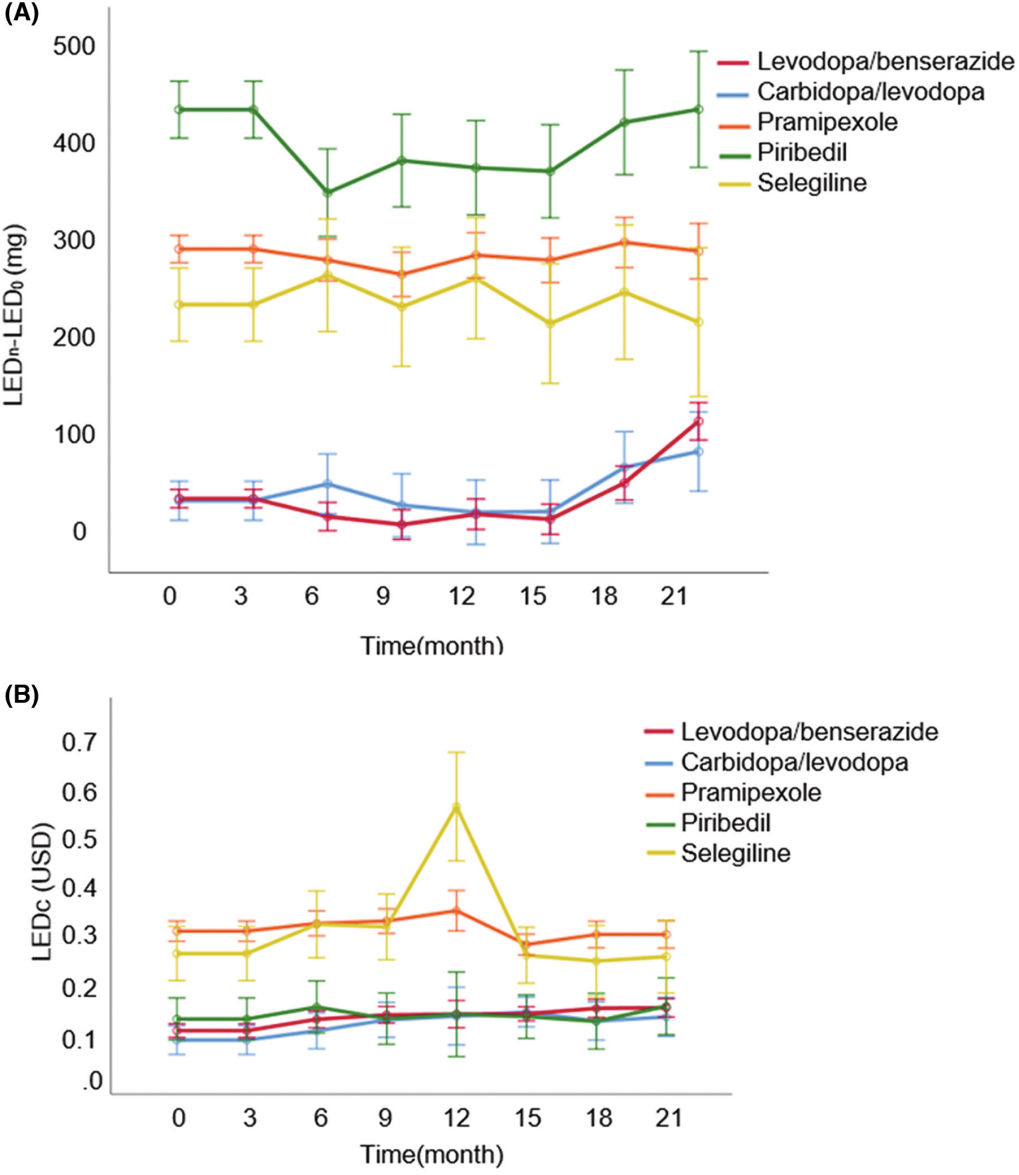
The trends of cumulative levodopa equivalent dose (LEDs) and levodopa equivalent dose cost (LEDc) over time. The gaps in the cumulative LEDs between every 3 months and baseline for each anti‐Parkinson drug (APD) group were presented by the mean ± SEM (mg) (A). The cumulative LEDc every 3 months of each APD group was presented by the mean ± SEM (USD) (B).

The effectiveness of different APDs was compared by the change trends of cumulative LEDs for each treatment group in every 3 months. Patients treated with piribedil possessed higher overall ΔLEDs values in all the follow‐up periods, followed by pramipexole and selegiline, carbidopa/levodopa, and levodopa/benserazide (Figure 2A).

For overall comparisons (Table 2), significant differences of ΔLEDs were found among APDs and in each group over time (group: *F* = 122.214, *p* < 0.001; time: *F* = 6.581, *p* < 0.001), while there was no significant difference in the interaction between group and time (time × group: *F* = 0.558, *p* = 0.971), indicating the potential intergroup variations of clinical improvements in response to different treatments as well as variations within each group over time, but not the interaction between the groups and times.

**TABLE 2:** Repeated measurement of ANOVA for ΔLEDs in overall comparison and pairwise comparison.

Factors	*F*		*p*
Time	6.581		<0.001
Group	122.214		<0.001
Time × Group	0.558		0.971

	Difference of ΔLEDs between two groups (mean ± SEM, mg)	95% CI	*p*
Levo/Ben versus Car/Levo	−5.32 ± 26.79	(−57.96, 47.31)	0.843
Levo/Ben versus Pra	−242.48 ± 20.48***	(−282.712, −202.24)	<0.001
Levo/Ben versus Piri	−355.04 ± 37.26***	(−428.25, −281.84)	<0.001
Levo/Ben versus Sele	−196.59 ± 46.92***	(−288.78, −104.40)	<0.001
Car/Levo versus Pra	−237.15 ± 29.58***	(−295.28, −179.02)	<0.001
Car/Levo versus Piri	−349.72 ± 42.94***	(−434.09, −265.34)	<0.001
Car/Levo versus Sele	−191.26 ± 51.55***	(−292.55, −89.98)	<0.001
Pra versus Piri	−112.56 ± 39.31**	(−189.82, −35.31)	0.004
Pra versus Sele	45.89 ± 48.57	(−49.55, 141.33)	0.345
Piri versus Sele	158.45 ± 57.69**	(45.10, 271.80)	0.006

*Note*: Significant difference: *p < 0.05, **p < 0.01, ***p < 0.001.

Abbreviations: Car/Levo, carbidopa/levodopa group; CI, confidence interval; Levo/Ben, levodopa/benserazide group; Piri, piribedil group; Pra, pramipexole group; Sele, selegiline group.

For group pairwise comparison, patients treated with carbidopa/levodopa or levodopa/benserazide had similar ΔLEDs (difference between carbidopa/levodopa and levodopa/benserazide (mean ± SEM): 5.32 ± 26.79 mg; *p* = 0.843). The ΔLEDs of these two groups were lower than those in pramipexole, piribedil, or selegiline groups, with the differences in ΔLEDs ranging around −191.26 to −355.04 mg, and the differences were statistically significant (*p* < 0.001). Moreover, the piribedil group had significantly higher ΔLEDs than both pramipexole and selegiline groups, while no significant difference was observed between pramipexole and selegiline (the differences of ΔLEDs between piribedil and pramipexole as 112.56 mg, *p* = 0.004; piribedil vs. selegiline as 158.45 mg, *p* = 0.006; pramipexole vs. selegiline as 45.89 mg, *p* = 0.345). Those results suggested that PD patients receiving levodopa/benserazide or carbidopa/levodopa treatments might obtain the best improvement in clinical symptoms among all five groups with the lowest ΔLEDs values, followed by pramipexole and selegiline, and lastly the piribedil group.

### 3.3 Comparisons of costs for APDs

The medical costs of the treatments with different APDs were compared by the trends of cumulative LEDc for each treatment group in every 3 months. Patients treated with pramipexole or selegiline exhibited higher cumulative LEDc values than the other three groups in all the follow‐up periods (Figure 2B).

For overall comparisons, significant differences in LEDc were found among these five groups (group: *F* = 24.375, *p* < 0.001) (Table 3). However, the LEDc did not change with time, and the effects of the interaction between each group and time on LEDc were similar (time: *F* = 0.439, *p* = 0.878; time × group: *F* = 0.827, *p* = 0.724), manifesting that the financial burden incurred from APDs varied among intergroups whereas no intragroup difference was found over time.

**TABLE 3:** Repeated measurement of ANOVA for LEDc in overall comparison and pairwise comparison.

Factors	*F*		*p*
Time	0.439		0.878
Group	24.375		<0.001
Time × Group	0.827		0.724

	Difference of LEDc between two groups (mean ± SEM, USD)	95% CI	*p*
Levo/Ben versus Car/Levo	0.014 ± 0.035	(−0.054, 0.083)	0.676
Levo/Ben versus Pra	−0.180 ± 0.026***	(−0.232, −0.128)	<0.001
Levo/Ben versus Piri	−0.004 ± 0.048	(−0.099, 0.090)	0.927
Levo/Ben versus Sele	−0.178 ± 0.061**	(−0.298, −0.058)	0.004
Car/Levo versus Pra	−0.194 ± 0.038***	(−0.270, −0.119)	<0.001
Car/Levo versus Piri	−0.019 ± 0.056	(−0.128, 0.090)	0.733
Car/Levo versus Sele	−0.193 ± 0.067**	(−0.324, −0.061)	0.004
Pra versus Piri	0.176 ± 0.051**	(0.075, 0.276)	0.001
Pra versus Sele	0.002 ± 0.063	(−0.122, 0.126)	0.977
Piri versus Sele	−0.174 ± 0.075*	(−0.321, −0.026)	0.021

*Note*: Significant difference: *p < 0.05, **p < 0.01, ***p < 0.001.

Abbreviations: Car/Levo, carbidopa/levodopa group; CI, confidence interval; Levo/Ben, levodopa/benserazide group; Piri, piribedil group; Pra, pramipexole group; Sele, selegiline group.

In a pairwise comparison of different treatment groups, LEDc in levodopa/benserazide, carbidopa/levodopa, and piribedil groups were remarkably lower than those in pramipexole or selegiline groups ($0.088–0.135/day for levodopa/benserazide; $0.070–0.126/day for carbidopa/levodopa; $0.112–0.138/day for piribedil; $0.290–0.332/day for pramipexole; $0.229–0.544/day for selegiline; *p* < 0.01) while LEDc within these two subgroups was comparable (levodopa/benserazide vs. carbidopa/levodopa vs. piribedil: *p* = 0.676, 0.927, and 0.733; pramipexole vs. selegiline: *p* = 0.977). These results implied that patients treated with levodopa/benserazide, carbidopa/levodopa, or piribedil probably took less costs derived from APDs than those treated with pramipexole or selegiline.

### 3.4 Comparisons of adverse events

#### 3.4.1 Total adverse events

The most common adverse events of different APDs were evaluated by the additional diagnostics, complications, and corresponding occurrence time points, including gastrointestinal symptoms, neuropsychiatric symptoms, headache/dizziness, and abnormal blood pressure.

The total adverse event rate was 22.7% during the 21‐month follow‐up period. Over 35% of patients with piribedil were newly diagnosed with the above complications, followed by levodopa/benserazide, selegiline, carbidopa/levodopa, and pramipexole by rank, with incidence rates of 25.6%, 23.5%, 23.3%, and 16.4%, respectively. Abnormal blood pressure and neuropsychiatric symptoms were the most common adverse events, with total rates of 10.9% and 10.5%, followed by headache/dizziness symptoms (3.8%) and gastrointestinal adverse events (3.2%). The results indicated that pramipexole, carbidopa/levodopa, and selegiline might exhibit a lower risk in these four aspects of adverse effects, while adverse events of APDs appeared to predominantly include neuropsychiatric symptoms and abnormal blood pressure.

#### 3.4.2 Adverse events in different human body systems

**FIGURE 3:**
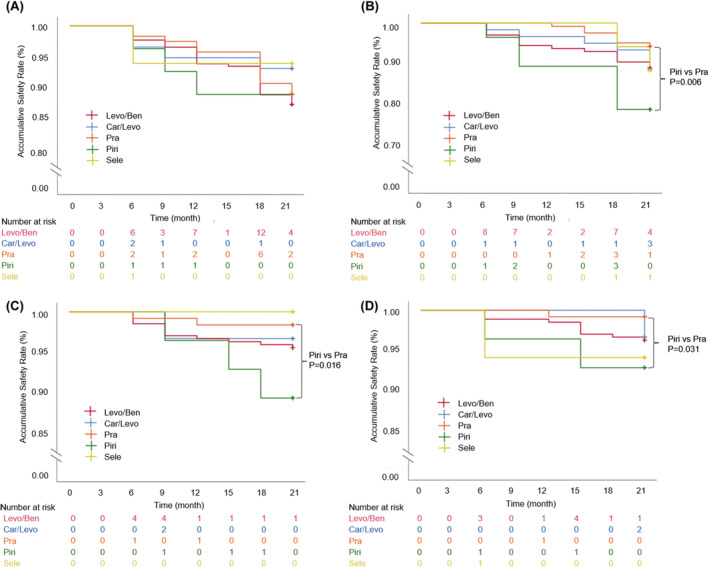
Adverse events‐free survival for patients with different anti‐Parkinson drugs (APDs). Kaplan–Meier curves with 21‐month landmark analysis for (A) abnormal blood pressure‐free events, (B) neuropsychiatric symptom‐free events, (C) headache/dizziness symptom‐free events, and (D) gastrointestinal symptom‐free events. The differences were determined using a log‐rank test. Car/Levo, carbidopa/levodopa group; Levo/Ben, levodopa/benserazide group; Piri, piribedil group; Pra, pramipexole group; Sele, selegiline group.

The total incidence of abnormal blood pressure was 10.9%. During the whole study period, 33 patients (12.3%) suffered from abnormal blood pressure in the levodopa/benserazide group with the highest incidence rate, followed by pramipexole and piribedil groups with the same incidence rate of 10.7%, and carbidopa/levodopa and selegiline groups had a lower incidence of around 6% (Table S1). Simultaneously, blood pressure‐related events first occurred among these five groups at the same time point of the 6‐month follow‐up period (Figure 3A). Log‐rank analysis indicated that the differences in abnormal blood pressure incidences in both overall comparisons and pairwise comparisons were not statistically significant (*p*>0.05) (Table S2).

The total occurrence rate of neuropsychiatric adverse events was about 10.5%, among which the pramipexole group showed the lowest incidence of 5.7%, while the piribedil group had the highest adverse event incidence of 21.4%, and the other three groups had similar rates of around 11% during the follow‐up period (Table S3). The survival analysis results of neuropsychiatric symptom‐free events showed that neuropsychiatric events occurred in the selegiline group with the latest appearance at the 18‐month time point of follow‐up period, the pramipexole group at 12‐month, and the carbidopa/levodopa, levodopa/benserazide, piribedil groups at 6‐month (Figure 3B). The statistical studies revealed that no statistically significant differences in the overall comparison for adverse events‐free survival change were observed among five groups, whereas a dramatical difference (*p* = 0.006) was found between the pramipexole and piribedil groups in a pairwise comparison, manifesting the possible lower risk of pramipexole in neuropsychiatric events within intraclass dopamine agonists, especially when compared with piribedil (Table S4).

The new occurrence frequency of headache/dizziness symptoms during the 21‐month observation period was 3.8%. The incidence in the piribedil group (10.7%) was higher than those in other groups (~5%), while no occurrence was detected in the selegiline group (Table S5). The appearance time of headache/dizziness symptoms was at 6 months of treatment in the levodopa/benserazide and pramipexole groups, at 9 months in the carbidopa/levodopa and piribedil groups, and the symptoms did not occur in the selegiline group during the follow‐up period (Figure 3C). Similar to neuropsychiatric events, the paired comparison of headache/dizziness events‐free survival change between pramipexole and piribedil groups had significant differences (*p* = 0.016), but not in the overall comparison (*p* = 0.192) (Table S6). Those results indicated the potential of selegiline and pramipexole to be applied as comparatively safer APDs with fewer headache/dizziness symptoms.

The total incidence of gastrointestinal adverse events was 3.2% during the follow‐up period. Up to the 21‐month follow‐up, the pramipexole group exhibited the lowest incidence rate of 0.8%, followed by carbidopa/levodopa (3.3%) and levodopa/benserazide (3.7%), and piribedil and selegiline groups with the higher incidence rates of 7.2% and 5.9%, respectively (Table S7). The gastrointestinal adverse event‐free survival changes over time were compared among these five APD groups. The first occurrences of gastrointestinal‐related events were at 6 months of treatment in the levodopa/benserazide, piribedil, and selegiline groups, about 12 months for the pramipexole group, and 21 months for the carbidopa/levodopa group, respectively (Figure 3D). The difference in the overall comparison was not significant (*p* = 0.370) (Table S8). By pairwise comparison with each group, pramipexole and piribedil groups had significant differences (*p* = 0.031), and the rest had no significant differences (*p >* 0.05), implying the presumable lower risk in gastrointestinal adverse events of the other four drugs except for piribedil, similar to the situations in gastrointestinal adverse events and headache/dizziness symptoms.

## 4 DISCUSSION

We conducted a multi‐center retrospective study using real‐world data to objectively make a comprehensive evaluation and comparison of different APDs in terms of effectiveness, safety, and costs. Our study indicated that the effectiveness of levodopa/benserazide and carbidopa/levodopa appeared to be superior to pramipexole and selegiline, while piribedil might be the least effective. The safety profile was comparable among all groups except for the piribedil group, in which patients were identified with higher risks of headache/dizziness, and gastrointestinal and neuropsychiatric symptoms. Drugs with higher unit prices, such as pramipexole and selegiline, might bring greater burdens than the remaining three APDs.

Levodopa is an intervention that is widely used in clinical trials with good control of PD symptoms. The latest Chinese Guideline for Anti‐Parkinson's Disease (fourth edition) recommends levodopa as the prior and primary basis for PD therapy, which was also supported by the results of our research. We found that levodopa‐based preparations were the most frequent as well as the most efficient drugs when compared with other classes. Additionally, for interclass comparison of efficacy, consistent with our research, a Cochrane meta‐analysis including 44 trials involving 8436 participants has also revealed that as adjuvant treatments to levodopa therapy, dopamine agonists were more efficacious in PD patients with UPDRS score improvements, off‐time, and levodopa dose reduction than MAOBIs and COMTIs.^14^ While for the intraclass comparison between dopamine agonists, several network meta‐analyses have reported that pramipexole and piribedil had no significant differences in improving non‐motor symptoms with similar UPDRS II and UPDRS III scores in early Parkinson's disease,^26^, ^27^ which was not in line with our study. Meanwhile, pramipexole was likely superior to selegiline in effectiveness as monotherapy, which turned out to be the opposite when used in combination with levodopa.^28^ This inconsistent conclusion may be partially due to the difference in the efficacy assessment indexes (UPDRS score, while in this study it was LED) and the sample sizes of the pramipexole and piribedil groups in our study. The indirect comparison in the meta‐analysis might also result in bias or other uncertainties. Head‐to‐head randomized controlled trials (RCTs) are needed to thoroughly validate the conclusions, and different clinical symptoms or disease stages of PD should be considered.

With regard to the safety of different APDs, previous studies have also supported our findings of a high incidence of similar adverse events in PD patients treated with piribedil. A recent meta‐analysis adopting a mixed treatment comparison showed that piribedil was one of the APDs with relatively higher incidence rates of nausea, dyskinesia, hallucination, dizziness, constipation, and somnolence symptoms among 11 different drugs involving pramipexole and levodopa.^29^ Piribedil, as a non‐ergotamine dopamine D_2/3/4_ receptor agonist and alpha 2A/B/C blocker, is applied as a monotherapy or adjuvant to L‐DOPA in PD treatment.^30^ The high affinities for the dopamine D_2_ and D_3_ receptors of dopamine agonists, however, might result in impulse control disorders through D_3_ receptors. Dopamine agonists could cause side effects while acting anti‐Parkinson's functions.^31^ The relatively strong anticholinergic properties of piribedil may be the underlying mechanism contributing to its high incidence of dizziness. Especially when piribedil is used in a long‐term manner, other potential anticholinergic side effects may develop, such as hallucinations, sicca symptoms, tachycardia, and urinary difficulty.^32^ Noteworthy, piribedil might induce even worse levodopa‐associated dyskinesia in a pre‐clinical study.^33^ Consistently, the increase in the overall incidence of side effects was generally more marked with dopamine agonists (OR 1.52) than with MAOBIs (OR 1.32), although heterogeneity between drug classes was only of borderline significance (*p* = 0.07).^14^

PD is the fourth most costly neurological disease after migraine, stroke, and epilepsy. Drugs for managing PD remained the major burden source, which accounted for 60.48% of the cost per outpatient visit, while medications for managing complications of PD weighted a smaller proportion.^24^, ^34^ Therefore, evaluating and comparing the costs of different APDs are of great significance. Our earlier single‐center studies have reported that the average cost of pramipexole per outpatient visit or admission episode was the highest among different medications, followed by selegiline, piribedil, levodopa/benserazide, and carbidopa/levodopa, but the previous studies lacked comparative statistics to conduct the comparison of therapeutic drugs.^24^ The conclusion was proved with consistent results in our expanded multiple‐center study and further improved to compare the costs among different APDs. Lin et al. found that levodopa/benserazide had obvious economic advantages when compared with benzhexol hydrochloride and pramipexole in early PD.^35^ A recent retrospective study analyzing the costs of APDs from 30 tertiary hospitals in China between 2014 and 2019 also indicated the highest average daily treatment cost of pramipexole with an average DDDc of $7.284/day and the relatively low DDDc in levodopa/carbidopa, selegiline, and levodopa/benserazide of $0.513, $0.542, and $0.641/day, respectively.^36^ Similar results were also drawn by an open‐label randomized and controlled trial in the UK, showing that the total medical costs of MAOBIs were lower than those of DAs while their quality‐adjusted life years were approximate.^37^ However, in our study, selegiline had a similar LEDc with that of pramipexole, and these distinct results might be attributed to the variation in the daily and total doses of MAOBIs or DAs as well as the treatment patterns. Moreover, treatment time was also regarded as an important factor affecting drug costs. For instance, pramipexole was proved unlikely to have cost‐effectiveness in 1–2 years compared with levodopa/carbidopa.^38^ Nevertheless, the probability of pramipexole being cost‐effective increased over time when the follow‐up time was extended from 2 to 4 years.^39^ Cost‐effectiveness should be considered and dynamically compared in different APDs with time.

Currently, there are very limited direct head‐to‐head RCTs about integrated evaluation for the efficacy, safety, and costs of intraclass, especially interclass, APDs. Even though indirect comparisons have been reported in several meta‐analysis studies, those results should be interpreted cautiously, as differences in the trial populations, protocol‐specified doses, and outcome measures assessed can introduce spurious differences between drug classes. To our knowledge, this is the first multi‐center retrospective study to systematically evaluate and compare the effectiveness, safety, and costs of different APDs. Our study fills the knowledge gap of the limited study about comprehensive intraclass and interclass comparisons of the effectiveness, safety, and economic benefits of different APDs, which provided evidence for practical decision‐making. Moreover, given the properties of chronic disease progression and the relatively slow action of APDs, most patients with PD usually initiate or adjust their treatments in outpatient rather than inpatient service, making the detailed information collection and the direct evaluations of effectiveness and safety in APDs more difficult. To overcome that, our study first utilizes outpatient prescription data to deduce the efficacy and adverse events of APDs. We conducted intraclass and interclass comparisons of multiple interventions, despite their complexity. This study might provide a rapid and simple evaluation method for APDs, which still needs further research for verification. In addition, the data source was from the electronic medical records of 30 tertiary hospitals for five consecutive years, which might have improved the accuracy of the results and the representativeness of the data. This also indicates that the findings in this study may be generalized to the greater population of China.

Several limitations of this study should be noted. First, since this study was conducted by analyzing the medical records in outpatient prescriptions, there was no direct data about drug response or tolerability for measuring the effectiveness and safety of APDs. We assessed effectiveness based on dosage changes for APDs and defined complications after PD diagnosis as PD‐related adverse events. It could lead to an underestimation or overestimation of the safety of APDs, since it is hard to distinguish whether the complications were directly attributable to adverse events of APDs. Additionally, adverse events may not be fully recorded in the prescriptions. Second, the disease stages and severity of PD might be incomparable among groups, as indicated by the differences in the intergroup LEDs at baseline. The disease stages of PD could relate to the absolute dosages of LEDs and incidences of complications. We deducted the baseline by calculating the gaps of LEDs between each time node and its starting time node to minimize the impacts incurred from different grading PD stages. Third, the adopted data necessarily assumes that every prescription represents medication consumed by the patient; in reality, the patient may not have visited the doctor to receive a prescription every time, or the prescription may not contain every drug ingested by patients. In this study, it is reasonable to suppose that the occurrences of medication switching or dosage adjustment in hospitals and the missing values were imputed by using a last‐observation‐carried‐forward approach. Finally, this study has only taken the total direct medical costs from APDs into account rather than all‐cause healthcare costs associated with PD treatment, which cannot fully reflect the economic impact of diseases on patients and health systems. Despite these limitations, medical records on outpatient prescriptions have been demonstrated to be a valid measure of patients' exposure to their medications. Moreover, in the next step, we will design and conduct prospective cohort studies on inpatients with PD to make up for the above limitations and further verify our research.

## 5 CONCLUSIONS

Overall, this multiple‐center retrospective study suggests that levodopa‐based preparations might exhibit better clinical improvement with fewer costs derived from APDs than pramipexole or selegiline, and piribedil presents less clinical improvement but a higher risk of headache/dizziness, and gastrointestinal and neuropsychiatric symptoms. More multi‐center, prospective, high‐quality RCTs, and cohort studies are needed in the future to verify our research and further reveal other in‐depth discoveries.

## AUTHOR CONTRIBUTIONS

All the authors contributed extensively to the work presented in this paper. Wenting Li conducted the literature review, study design, data analysis, and manuscript preparation. Hua Zhang guided the statistical analysis of the data. Yuan Zhang provided suggestions and participated in the manuscript revision. Ke Wang and Jiaojiao Hui contributed to the result interpretation and manuscript writing. Zhanmiao Yi obtained data, designed and supervised the whole research activities, revised the manuscript, and obtained the funding for the study. All authors have read and approved the final manuscript.

## FUNDING INFORMATION

This study was supported by the National Natural Science Foundation of China (72104003).

## CONFLICT OF INTEREST STATEMENT

The authors have no conflicts of interest to disclose.

## Supporting information


Table S1.
Table S2.
Table S3.
Table S4.
Table S5.
Table S6.
Table S7.
Table S8.

## Data Availability

The data supporting the findings of this study are available from the corresponding author on reasonable request.
