# Supplementary material for: Comparison of the effectiveness, safety, and costs of anti‐Parkinson drugs: A multiple‐center retrospective study
Source: CNS Neurosci Ther. 2023 Nov 20;30(4):e14531. doi: 10.1111/cns.14531 (PMC11017413; doi:10.1111/cns.14531)
Supplement: Supplementary file 1 — Table S1. Table S2. Table S3. Table S4. Table S5. Table S6. Table S7. Table S8. [file CNS-30-e14531-s001.docx]

**Supplementary Data**

**Supplementary Table 1. Abnormal blood pressure-free events in each APDs treatment group over time.**

| Group | Safety events (n, %) | | | | | | | |
| --- | --- | --- | --- | --- | --- | --- | --- | --- |
|  | 0-month | 3-month | 6-month | 9-month | 12-month | 15-month | 18-month | 21-month |
| Levo/Ben | 269(100%) | 269(100%) | 263(97.8%) | 260(96.6%) | 253(94.0%) | 252(93.7%) | 240(89.2%) | 236(87.7%) |
| Car/Levo | 60(100%) | 60(100%) | 58(98.3%) | 57(95.0%) | 57(95.0%) | 57(95.0%) | 56(93.3%) | 56(93.3%) |
| Pra | 122(100%) | 122(100%) | 120(98.4%) | 119(97.5%) | 117(95.9%) | 117(95.9%) | 111(91.0%) | 109(89.3%) |
| Piri | 28(100%) | 28(100%) | 27(96.4%) | 26(92.8%) | 25(89.3%) | 25(89.3%) | 25(89.3%) | 25(89.3%) |
| Sele | 17(100%) | 17(100%) | 16(94.1%) | 16(94.1%) | 16(94.1%) | 16(94.1%) | 16(94.1%) | 16(94.1%) |

Abbreviations: Car/Levo, carbidopa/levodopa group; Levo/Ben, levodopa/benserazide group; Piri, piribedil group; Pra, pramipexole group; Sele, selegiline group.

**Supplementary Table 2. Log-rank test for** **abnormal blood pressure-free events in overall comparison and pairwise comparison.**

| Log Rank (Mantel-Cox) | Chi-square (X^2^) | *F* | *P* |
| --- | --- | --- | --- |
| Overall | 1.933 | 4 | 0.748 |
| Levo/Ben versus Car/Levo | 1.429 |  | 0.232 |
| Levo/Ben versus Pra | 0.232 |  | 0.630 |
| Levo/Ben versus Piri | 0.033 |  | 0.856 |
| Levo/Ben versus Sele | 0.562 |  | 0.454 |
| Car/Levo versus Pra | 0.681 |  | 0.409 |
| Car/Levo versus Piri | 0.421 |  | 0.517 |
| Car/Levo versus Sele | 0.011 |  | 0.915 |
| Pra versus Piri | 0.005 |  | 0.942 |
| Pra versus Sele | 0.332 |  | 0.565 |
| Piri versus Sele | 0.278 |  | 0.598 |

Abbreviations: Car/Levo, carbidopa/levodopa group; Levo/Ben, levodopa/benserazide group; Piri, piribedil group; Pra, pramipexole group; Sele, selegiline group.

**Supplementary Table 3. Neuropsychiatric symptom-free events in each APDs treatment group over time.**

| Group | Safety events (n, %) | | | | | | | |
| --- | --- | --- | --- | --- | --- | --- | --- | --- |
|  | 0-month | 3-month | 6-month | 9-month | 12-month | 15-month | 18-month | 21-month |
| Levo/Ben | 269(100%) | 269(100%) | 261(97.0%) | 254(94.4%) | 252(93.7%) | 250(92.9%) | 243(90.3%) | 239(88.8%) |
| Car/Levo | 60(100%) | 60(100%) | 59(98.3%) | 58(96.7%) | 58(96.7%) | 57(95.0%) | 56(93.3%) | 53(88.3%) |
| Pra | 122(100%) | 122(100%) | 122(100%) | 122(100%) | 121(99.2%) | 119(97.5%) | 116(95.1%) | 115(94.3%) |
| Piri | 28(100%) | 28(100%) | 27(96.4%) | 25(89.3%) | 25(89.3%) | 25(89.3%) | 22(78.6%) | 22(78.6%) |
| Sele | 17(100%) | 17(100%) | 17(100%) | 17(100%) | 17(100%) | 17(100%) | 16(94.1%) | 15(88.2%) |

Abbreviations: Car/Levo, carbidopa/levodopa group; Levo/Ben, levodopa/benserazide group; Piri, piribedil group; Pra, pramipexole group; Sele, selegiline group.

**Supplementary Table 4. Log-rank test for neuropsychiatric symptom-free events in overall comparison and pairwise comparison.**

| Log Rank (Mantel-Cox) | Chi-square (X^2^) | *F* | *P* |
| --- | --- | --- | --- |
| Overall | 6.975 | 4 | 0.137 |
| Levo/Ben versus Car/Levo | 0.004 |  | 0.947 |
| Levo/Ben versus Pra | 2.955 |  | 0.086 |
| Levo/Ben versus Piri | 2.511 |  | 0.113 |
| Levo/Ben versus Sele | 0.000 |  | 0.984 |
| Car/Levo versus Pra | 1.983 |  | 0.159 |
| Car/Levo versus Piri | 1.577 |  | 0.209 |
| Car/Levo versus Sele | 0.000 |  | 0.987 |
| Pra versus Piri | 7.615 |  | 0.006** |
| Piri versus Sele | 0.772 |  | 0.379 |
| Pra versus Sele | 0.822 |  | 0.365 |

Abbreviations: Car/Levo, carbidopa/levodopa group; Levo/Ben, levodopa/benserazide group; Piri, piribedil group; Pra, pramipexole group; Sele, selegiline group. Significant difference: **P < 0.01.

**Supplementary Table 5. Headache/dizziness symptom-free events in each APDs treatment group over time.**

| Group | Safety events (n, %) | | | | | | | |
| --- | --- | --- | --- | --- | --- | --- | --- | --- |
|  | 0-month | 3-month | 6-month | 9-month | 12-month | 15-month | 18-month | 21-month |
| Levo/Ben | 269(100%) | 269(100%) | 265(98.5%) | 261(97.0%) | 260(96.6%) | 259(96.3%) | 258(95.9%) | 257(95.5%) |
| Car/Levo | 60(100%) | 60(100%) | 60(100%) | 58(96.7%) | 58(96.7%) | 58(96.7%) | 58(96.7%) | 58(96.7%) |
| Pra | 122(100%) | 122(100%) | 121(99.2%) | 121(99.2%) | 120(98.4%) | 120(98.4%) | 120(98.4%) | 120(98.4%) |
| Piri | 28(100%) | 28(100%) | 28(100%) | 27(96.4%) | 27(96.4%) | 26(92.8%) | 25(89.3%) | 25(89.3%) |
| Sele | 17(100%) | 17(100%) | 17(100%) | 17(100%) | 17(100%) | 17(100%) | 17(100%) | 17(100%) |

Abbreviations: Car/Levo, carbidopa/levodopa group; Levo/Ben, levodopa/benserazide group; Piri, piribedil group; Pra, pramipexole group; Sele, selegiline group.

**Supplementary Table 6. Log-rank test for headache/dizziness symptom-free events in overall comparison and pairwise comparison.**

| Log Rank (Mantel-Cox) | Chi-square (X^2^) | *F* | *P* |
| --- | --- | --- | --- |
| Overall | 6.104 | 4 | 0.192 |
| Levo/Ben versus Car/Levo | 0.155 |  | 0.694 |
| Levo/Ben versus Pra | 1.918 |  | 0.166 |
| Levo/Ben versus Piri | 2.005 |  | 0.157 |
| Levo/Ben versus Sele | 0.777 |  | 0.378 |
| Car/Levo versus Pra | 0.530 |  | 0.467 |
| Car/Levo versus Piri | 1.886 |  | 0.170 |
| Car/Levo versus Sele | 0.574 |  | 0.449 |
| Pra versus Piri | 5.776 |  | 0.016* |
| Pra versus Sele | 0.280 |  | 0.597 |
| Piri versus Sele | 1.890 |  | 0.169 |

Abbreviations: Car/Levo, carbidopa/levodopa group; Levo/Ben, levodopa/benserazide group; Piri, piribedil group; Pra, pramipexole group; Sele, selegiline group. Significant difference: *P < 0.05.

**Supplementary Table 7. Gastrointestinal symptom-free events in each APDs treatment group over time.**

| Group | Safety events (n, %) | | | | | | | |
| --- | --- | --- | --- | --- | --- | --- | --- | --- |
|  | 0-month | 3-month | 6-month | 9-month | 12-month | 15-month | 18-month | 21-month |
| Levo/Ben | 269(100%) | 269(100%) | 266(98.9%) | 266(98.9%) | 265(98.5%) | 261(97.0%) | 260(96.6%) | 259(96.3%) |
| Car/Levo | 60(100%) | 60(100%) | 60(100%) | 60(100%) | 60(100%) | 60(100%) | 60(100%) | 58(96.7%) |
| Pra | 122(100%) | 122(100%) | 122(100%) | 122(100%) | 121(99.2%) | 121(99.2%) | 121(99.2%) | 121(99.2%) |
| Piri | 28(100%) | 28(100%) | 27(96.4%) | 27(96.4%) | 27(96.4%) | 26(92.8%) | 26(92.8%) | 26(92.8%) |
| Sele | 17(100%) | 17(100%) | 16(94.1%) | 16(94.1%) | 16(94.1%) | 16(94.1%) | 16(94.1%) | 16(94.1%) |

Abbreviations: Car/Levo, carbidopa/levodopa group; Levo/Ben, levodopa/benserazide group; Piri, piribedil group; Pra, pramipexole group; Sele, selegiline group.

**Supplementary Table 8. Log-rank test for gastrointestinal symptom-free events in overall comparison and pairwise comparison.**

| Log Rank (Mantel-Cox) | Chi-square (X^2^) | *F* | *P* |
| --- | --- | --- | --- |
| Overall | 4.274 | 4 | 0.370 |
| Levo/Ben versus Car/Levo | 0.027 |  | 0.870 |
| Levo/Ben versus Pra | 2.552 |  | 0.110 |
| Levo/Ben versus Piri | 0.786 |  | 0.375 |
| Levo/Ben versus Sele | 0.220 |  | 0.639 |
| Car/Levo versus Pra | 1.533 |  | 0.216 |
| Car/Levo versus Piri | 0.694 |  | 0.405 |
| Car/Levo versus Sele | 0.257 |  | 0.612 |
| Pra versus Piri | 4.677 |  | 0.031* |
| Pra versus Sele | 2.765 |  | 0.096 |
| Piri versus Sele | 0.024 |  | 0.878 |

Abbreviations: Car/Levo, carbidopa/levodopa group; Levo/Ben, levodopa/benserazide group; Piri, piribedil group; Pra, pramipexole group; Sele, selegiline group. Significant difference: *P < 0.05.
